# The impact mechanism of national forest park environment perception on mental health: a mediation analysis based on forest health activity participation

**DOI:** 10.3389/fpubh.2026.1691989

**Published:** 2026-03-12

**Authors:** Fei Meng, Shao Qing

**Affiliations:** 1 School of Culture and Tourism, Henan Institute of Economics and Trade, Zhengzhou, China; 2 School of Business and Logistics, Henan Institute of Economics and Trade, Zhengzhou, China

**Keywords:** environmental perception, forest therapy, health behavior, mental health, national forest parks, stress recovery

## Abstract

**Background:**

National forest parks represent natural ecosystems with demonstrated therapeutic potential for public mental health. As multifunctional wellness spaces, these protected areas contribute to emotional regulation, stress mitigation, and psychological wellbeing enhancement. Current research provides essential foundations for optimizing health landscape configurations through quantitative analysis of forest environmental factors and mental health outcomes.

**Methods:**

This study employed structural equation modeling to investigate mediating pathways through which national forest park environments influence mental health. Grounded in Stress Recovery Theory and Attention Restoration Theory, the analysis used a 2025 survey dataset comprising 618 valid responses from Lanzhou residents. Key environmental perception dimensions were operationalized as exogenous variables to examine their direct and indirect effects on mental health indicators.

**Results:**

(1) Environmental perception dimensions significantly predicted psychological wellbeing, with naturalness perception showing the strongest effect (β = −0.487, *p* < 0.001), followed by health facility (β = −0.296, *p* < 0.001), service quality (β = −0.124, *p* < 0.05), and tranquility perceptions (β = −0.108, *p* < 0.10); (2) forest therapy participation served as a significant mediator with indirect effects ranging from 0.144 to 0.243 (all *p* < 0.001); and (3) high-stress individuals demonstrated substantially stronger therapeutic benefits, with indirect effects 5.7–6.3 times larger than low-stress counterparts and significantly enhanced participation-wellbeing relationships (*p* < 0.001).

**Conclusion:**

The findings indicate substantial mental health benefits from national forest park environments. Strategic planning should prioritize: (1) enhancing naturalness through ecological restoration; (2) optimizing landscape configuration for sensory tranquility; and (3) expanding health-promoting infrastructure. These interventions may enhance ecosystem services in addressing urban mental health challenges.

## 1 Introduction

National forest parks, as an essential component of the natural protected area system, are defined as natural areas primarily focused on protecting forest ecosystems while possessing ecological, aesthetic, cultural, and scientific values for sustainable utilization (1, 2). They serve as the primary carriers of ecological welfare provision and forest therapy services. With the rapid advancement of urbanization and high-speed socioeconomic development, the fast-paced urban lifestyle and high-pressure work environments have made mental health issues among urban residents (3, 4) increasingly prominent. According to the “China National Mental Health Development Report (2021–2022),” depression and anxiety symptoms affect approximately 34.7% of Chinese adults, with urban residents showing higher prevalence rates than rural populations. Moreover, work-related stress has been identified as a primary contributor to psychological distress, particularly among mid-career professionals aged 30–45 years. Against this backdrop, national forest parks, as natural “green sanatoriums,” have gradually attracted widespread attention from government departments and scholars across various disciplines for their unique value in mental health promotion, emotion regulation, and stress relief (5, 6).

Current research on the health effects of forest environments is primarily based on Stress Recovery Theory (7–9) and Attention Restoration Theory (10, 11). Stress Recovery Theory posits that natural environments, particularly forest environments, can activate the human parasympathetic nervous system and reduce cortisol levels, thereby rapidly alleviating psychological stress and physiological tension (12–14). Attention Restoration Theory emphasizes that the “soft fascination” characteristics of forest environments can help individuals recover from directed attention fatigue, enhancing cognitive function and emotional states (11, 15).

There remains a need for deeper exploration of how individuals' multi-sensory experiences of forest environments influence their mental health (16). The impact of forest environments on individual psychological states varies according to personal internal factors and external environmental factors (17). On one hand, high-quality forest environments stimulate positive public perception, enhance therapy experience satisfaction, and generate sustained health behaviors; on the other hand, unsuitable forest characteristics and individual barrier factors also affect the realization of therapy effectiveness (18–21).

Theoretical foundations for mediation and moderation pathways warrant explicit elaboration. According to the Theory of Planned Behavior (16), environmental perceptions shape behavioral intentions before influencing health outcomes, establishing the theoretical rationale for mediation through activity participation. This sequential process—from perception to behavior to health—has been empirically supported in nature-based health research (17, 22), wherein environmental quality assessments first enhance participation motivation, which subsequently improves psychological wellbeing.

The moderation framework is grounded in Stress Recovery Theory (7, 9), which posits differential restorative responses based on baseline stress levels. High-stress individuals possess greater “restorative deficit,” theoretically predicting enhanced sensitivity to therapeutic environments (13). This stress-dependent response has been documented in environmental psychology (23), where individual characteristics moderate both the perception-behavior relationship and the behavior-outcome relationship. Specifically, stress level may amplify the effect of environmental perception on participation willingness (first-stage moderation) and strengthen the therapeutic benefits derived from participation (second-stage moderation).

Despite these theoretical foundations, integrated frameworks simultaneously examining mediation and moderation mechanisms within forest therapy contexts remain limited. Most studies have tested either mediation or moderation separately (22, 23), without investigating how individual differences (stress levels) conditionally influence the indirect pathways through which environmental factors affect mental health.

**Figure 1:**
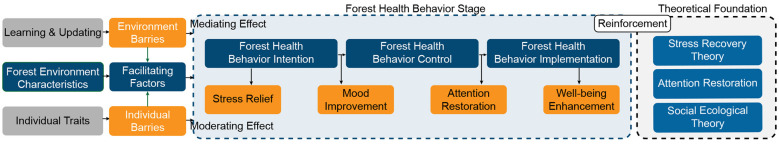
Theoretical model of forest-based health behavior: the mediating role of behavioral participation and moderating effects of individual characteristics.

This study addresses these research gaps by examining the mechanisms through which forest environment perception influences psychological wellbeing. As illustrated in Figure 1, the investigation focuses on: (1) the multidimensional construct of forest environment perception, operationalized as first-order latent variables encompassing naturalness, tranquility, health facility accessibility, and service quality; (2) the mediation effect of forest therapy activity participation (22) on the perception–wellbeing relationship; and (3) the moderating influence of individual stress levels on this mediation pathway.

Based on the theoretical foundations and empirical evidence, we propose the following hypotheses:

H1: Forest environment perception dimensions (naturalness, tranquility, health facility, and service quality) are negatively associated with psychological distress.

H2: Forest therapy activity participation mediates the relationship between environmental perception and psychological wellbeing.

H3: Individual stress level moderates the relationship between environmental perception and forest therapy activity participation, such that the positive association is stronger for high-stress individuals.

H4: Individual stress level moderates the relationship between forest therapy activity participation and psychological wellbeing, such that the negative association (therapeutic effect) is stronger for high-stress individuals.

## 2 Study area and research methods

### 2.1 Study area overview and data sources

**Figure 2:**
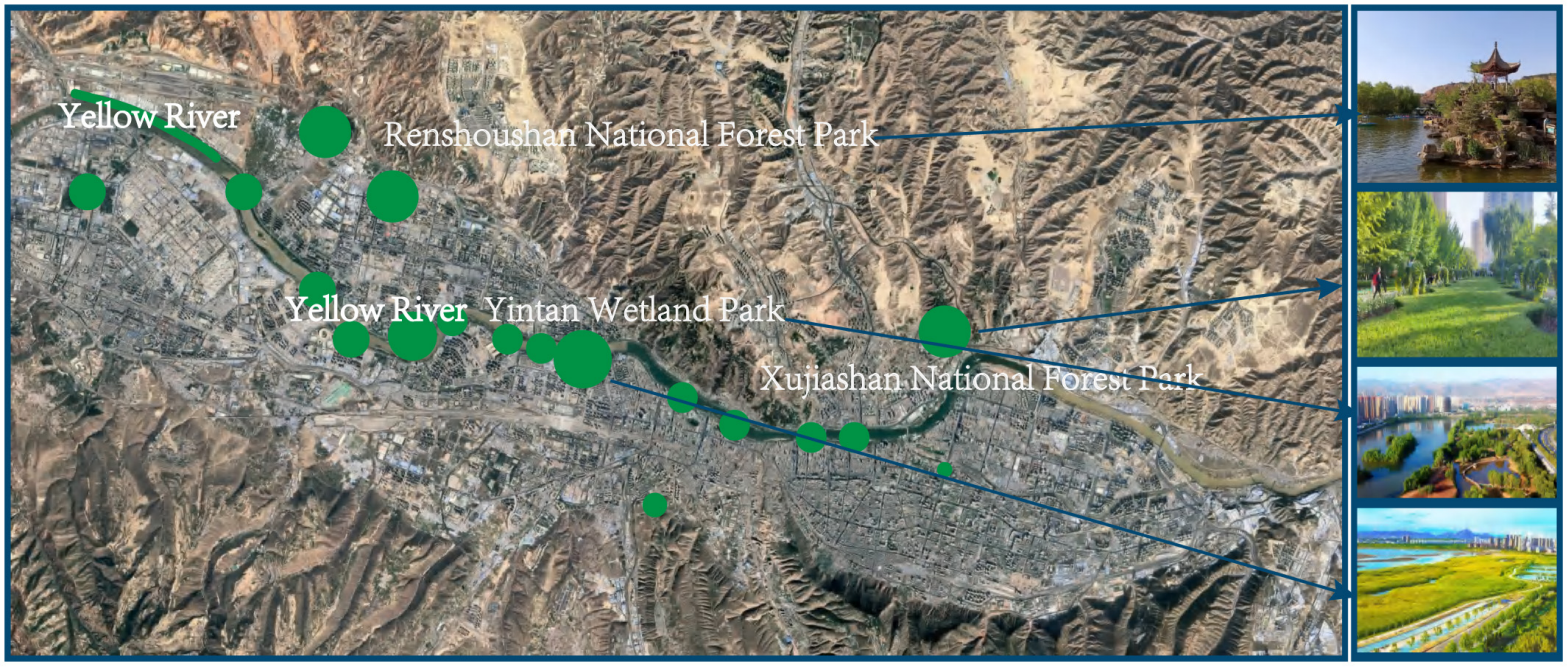
Major urban green spaces in Lanzhou downtown area and the (three of four) urban parks selected in our study.

Lanzhou City is located in northwestern China (36°03′N, 103°40′E), serving as the capital of Gansu Province (24). This study selected three national forest parks within Lanzhou metropolitan area: Renshoushan National Forest Park, Xujiashan National Forest Park, and Yellow River-Yintan Wetland Park (Figure 2). These parks exhibit typical temperate continental climate characteristics with forest coverage exceeding 75%, providing ideal settings for investigating forest environment effects on mental health.

Data collection and quality control: primary data were collected through structured online surveys from March to June 2025. The target population comprised residents who had visited the three forest parks for health-promoting activities within the past 30 days. Due to practical constraints, a convenience sampling approach was adopted, whereby respondents were recruited through online platforms and social media groups associated with the three parks. While this method facilitated efficient data collection, it may limit the generalizability of findings to the broader population.

Multiple quality assurance measures were implemented: survey administration was conducted using a professional online platform (Wenjuanxing, https://www.wjx.cn/) with multiple quality assurance measures implemented: (1) attention check questions: systematic insertion of attention detection items at randomized positions to verify respondent engagement; (2) response consistency verification: logic validation algorithms to identify contradictory responses across related items; (3) response time analysis: elimination of surveys completed in unreasonably short durations (< 5 min) or excessively long periods (>45 min); (4) geographic validation: IP address verification to confirm respondents' location within Lanzhou metropolitan area; and (5) duplicate response prevention: unique device fingerprinting to prevent multiple submissions from the same respondent. Following data cleaning procedures, 618 valid responses were retained from 847 initial submissions (validity rate: 73.0%). The final sample showed balanced representation across parks: Renshoushan (37.9%), Xujiashan (31.6%), and Yellow River-Yintan (30.6%).

### 2.2 Scale and questionnaire design

#### 2.2.1 Independent variable measurement items

Based on relevant research on forest therapy environmental perception (20, 21), this study considers that national forest park environmental perception primarily includes four dimensions: naturalness perception, tranquility perception, health facility perception, and service perception. Drawing from measurement scales for natural environment perception by (25, 26), while referencing research methods by (27, 28), and combining the environmental characteristics of national forest parks, a forest environmental perception scale was designed (Table 1).

**Table 1:** Measurement instruments and psychometric properties.

**Construct**	**Measurement scale**	**Items**	**Scale type**	**Cronbach's α**
**Independent variable: forest environment perception**				
Naturalness perception	Adapted from Balram et al. (25)	4	5-point Likert	0.85
Tranquility perception	Adapted from Grahn and Stigsdotter (39)	4	5-point Likert	0.82
Health facility perception	Adapted from Ugolini et al. (40)	4	5-point Likert	0.78
Service quality perception	Self-developed	4	5-point Likert	0.75
**Mediating variable**				
Forest therapy activity	Self-developed	4	5-point frequency	0.82
participation			(never to always)	
**Dependent variable**				
Psychological wellbeing	GHQ-12 (41)	12	5-point Likert	0.79
**Moderating variable**				
Individual stress level	PSS-10 (42)	10	5-point Likert	0.83
**Control variables**				
Demographics	–	6	Various	–

All scales demonstrated satisfactory convergent validity (AVE>0.5) and discriminant validity.

GHQ-12, 12-item General Health Questionnaire; PSS-10, 10-item Perceived Stress Scale.

The scale development process involved three stages: (1) initial item pool generation: based on literature review and expert consultation, 20 preliminary items were generated across four dimensions; (2) expert evaluation: five experts in environmental psychology, forest therapy, and landscape architecture reviewed the items for content validity. Items with poor relevance or redundancy were removed, resulting in 16 items (four per dimension); and (3) pilot testing: a pilot survey with 85 respondents (mean age = 32.4 years, SD = 8.7; 52.9% female) was conducted in February 2025 to assess psychometric properties. Cronbach's alpha coefficients ranged from 0.73 to 0.84 across dimensions, and exploratory factor analysis confirmed the four-factor structure (cumulative variance explained = 68.3%). Based on participant feedback and item-total correlations, minor wording adjustments were made to enhance clarity while preserving construct validity.

The final scale includes the following specific items:

Naturalness perception (four items): (1) the forest park has abundant and diverse vegetation species; (2) the air quality in the forest park is fresh and comfortable; (3) the natural landscape in the forest park is well-preserved and authentic; and (4) the biodiversity in the forest park is rich and vibrant.

Tranquility perception (four items): (1) the forest park provides a quiet environment away from urban noise; (2) the natural sounds in the forest park are pleasant and relaxing; (3) the forest park offers sufficient space for peaceful contemplation; and (4) the overall atmosphere in the forest park is calm and serene.

Health facility perception (four items): (1) the forest park has well-maintained walking and hiking trails; (2) rest facilities such as benches and shelters are adequately provided; (3) health-promoting facilities meet visitors' therapeutic needs; and (4) the layout of facilities is convenient and accessible.

Service quality perception (four items): (1) park management staff are friendly and helpful; (2) visitor information and guidance services are clear and adequate; (3) the park is well-maintained with regular cleaning and upkeep; and (4) safety measures and emergency services are satisfactory.

A 5-point Likert scale (29) was used to evaluate each measurement item, with each item having five response options: “strongly agree,” “somewhat agree,” “uncertain,” “somewhat disagree,” and “strongly disagree,” scored as 5, 4, 3, 2, and 1 points, respectively. Upon testing, the Cronbach's α coefficient of this scale ranged from 0.75 to 0.85, indicating good reliability.

#### 2.2.2 Mediating variable measurement items

The mediating variable in this study is forest therapy activity participation. Based on the characteristics of forest therapy, measurements were conducted across aspects including forest bathing activities, forest walking, forest meditation, and nature observation (Table 1). The specific items are: (1) I engage in forest bathing or breathing exercises in the forest park; (2) I participate in forest walking or hiking activities in the forest park; (3) I practice meditation or mindfulness exercises in the forest park; and (4) I engage in nature observation or nature appreciation activities in the forest park. A 5-point positive scoring system was used where “never participate,” “rarely participate,” “occasionally participate,” “frequently participate,” and “always participate” were scored 1–5 points, respectively, with higher scores indicating higher individual forest therapy activity participation. Upon testing, the Cronbach's α coefficient of the forest therapy activity participation scale was 0.815, indicating good internal consistency.

#### 2.2.3 Dependent variable measurement items

For the dependent variable of mental health, the internationally recognized GHQ-12 (the 12-item form of the General Health Questionnaire) (30) scale was used for measurement. This scale has been widely used in research on mental health effects of natural environments and has been proven to have good validity and reliability, with specific measurement items shown in Table 1. The scale scoring employed a 5-point scoring method. Items 3–6 and items 8–9 were positive items, while items 1, 2, 7, 10, and 12 were negative items. For positive items, reverse scoring was used, with scores from 5 to 1 representing respondents' choices of “not at all,” “same as usual,” “uncertain,” “rather more than usual,” and “much more than usual”; for negative items, forward scoring was used, with scores from 1 to 5 representing “not at all,” “same as usual,” “uncertain,” “rather more than usual,” and “much more than usual.” Lower scale scores indicated higher mental health levels among respondents. The Cronbach's α (31) coefficient of this scale in this study was 0.79.

#### 2.2.4 Moderating variable measurement items

Individual stress level was included as a moderating variable based on theoretical and empirical considerations. According to Stress Recovery Theory (7, 9), individuals experiencing higher stress have greater restorative needs and may be more sensitive to environmental therapeutic effects. Previous research has demonstrated that baseline stress levels moderate the relationship between natural environment exposure and health outcomes (22, 23), with high-stress individuals showing stronger therapeutic responses. Therefore, examining stress as a moderator provides insights into differential treatment effects across population subgroups.

A simplified version of the Perceived Stress Scale (PSS-10) (32) was employed to measure individual stress conditions across aspects including work stress, life stress, and economic stress, using a 5-point scoring system where higher scores indicated higher stress levels. Respondents were divided into high-stress and low-stress groups based on median split to examine differences in forest therapy effects among populations with different stress levels.

#### 2.2.5 Questionnaire design

To ensure scale validity, this study semantically examined the internal consistency of measurement items for each factor. Additionally, experts in forest therapy, environmental psychology, and public health were invited to evaluate whether the measurement items could represent the measured constructs and whether the measurement items could cover the measurement scope. Finally, the above scales were compiled into a questionnaire and a pilot survey was conducted on a small scale to calculate the internal consistency of each scale.

Confirmatory Factor Analysis (CFA) was conducted to assess the measurement model's validity. The CFA model demonstrated acceptable fit indices: χ^2^/df = 2.34, CFI = 0.94, TLI = 0.93, RMSEA = 0.061 (90% CI [0.055, 0.067]), SRMR = 0.048. All factor loadings exceeded 0.60 and were statistically significant (*p* < 0.001), supporting construct validity.

Reliability and validity metrics are presented in Table 2. Composite Reliability (CR) values ranged from 0.76 to 0.86, exceeding the recommended threshold of 0.70. Average Variance Extracted (AVE) values ranged from 0.52 to 0.64, all above 0.50, indicating satisfactory convergent validity. Discriminant validity was assessed using the Fornell–Larcker criterion (Table 3). As shown in the correlation matrix, the square root of AVE for each construct (diagonal values: 0.72–0.80) exceeded all inter-construct correlations (off-diagonal values: 0.22–0.68), confirming adequate discriminant validity across all measurement scales.

**Table 2:** Reliability and validity analysis results.

**Construct**	**Items**	**CR**	**AVE**	** $\sqrt{\text{AVE}}$ **	**Factor loading range**
Naturalness perception	4	0.86	0.64	0.80	0.72–0.85
Tranquility perception	4	0.83	0.58	0.76	0.68–0.81
Health facility perception	4	0.79	0.52	0.72	0.63–0.78
Service quality perception	4	0.76	0.53	0.73	0.65–0.79
Forest therapy participation	4	0.83	0.56	0.75	0.67–0.82
Psychological wellbeing	12	0.81	0.54	0.73	0.61–0.77
Individual stress level	10	0.84	0.55	0.74	0.62–0.80

All factor loadings are significant at *p* < 0.001. Recommended thresholds: CR > 0.70, AVE > 0.50.

CR, composite reliability; AVE, average variance extracted.

**Table 3:** Discriminant validity: correlation matrix and square root of AVE.

**Construct**	**1**	**2**	**3**	**4**	**5**	**6**	**7**
1. Naturalness perception	**0.80**						
2. Tranquility perception	0.68	**0.76**					
3. Health facility perception	0.64	0.59	**0.72**				
4. Service quality perception	0.61	0.56	0.67	**0.73**			
5. Forest therapy participation	0.58	0.52	0.54	0.49	**0.75**		
6. Psychological wellbeing	–0.52	–0.47	–0.48	–0.43	–0.51	**0.73**	
7. Individual stress level	0.28	0.26	0.24	0.22	0.31	0.64	**0.74**

Diagonal elements (in bold) represent the square root of AVE. Off-diagonal elements represent inter-construct correlations. All correlations are significant at *p* < 0.01. Discriminant validity is established when diagonal values exceed off-diagonal values in the same row/column.

### 2.3 Research model

#### 2.3.1 Theoretical foundation and model construction

We propose a theoretical framework for understanding the mental health promotion mechanisms of forest therapy by integrating Stress Recovery Theory (SRT) (7–9) and Attention Restoration Theory (ART) (10, 11), as illustrated in Figure 1. According to SRT, natural environments exhibit distinct restorative properties that reduce physiological and psychological stress through parasympathetic nervous system activation (33). ART further identifies four key restorative attributes of natural settings—being away, fascination, extent, and compatibility—which synergistically enhance attention restoration capacities (34).

Building upon Social Ecological Theory (SET) (35), this research conceptualizes the mental health benefits of forest therapy as an outcome of dynamic interactions among three interrelated domains: individual characteristics, behavioral mediators, and environmental conditions. Specifically, forest environmental perception—operationalized as individuals' subjective evaluation of ecological attributes—serves as a critical antecedent influencing health-promoting behaviors (behavioral mediators). Concurrently, individual stress levels (individual characteristics) function as a moderating variable that regulates the strength and direction of these behavioral-environmental interactions.

#### 2.3.2 Mediation effect model

Based on the mediation effect testing framework proposed by (22), a mediation effect model was constructed with forest environmental perception as the independent variable, forest therapy activity participation as the mediating variable, and mental health as the dependent variable. According to the mechanisms of forest therapy, individuals' positive perception of forest environments promotes their participation in various forest therapy activities, while participation in health activities directly influences mental health levels. The specific model specifications are as follows:

Step 1: Testing total effect


$$\begin{array}{l} Y=c_{0}+cX+\overset{n}{\underset{i=1}{\sum }}\gamma_{i}Controls_{i}+\varepsilon_{1} \end{array}$$ (1)

Step 2: Testing first-half path


$$\begin{array}{l} M=a_{0}+aX+\overset{n}{\underset{i=1}{\sum }}\delta_{i}Controls_{i}+\varepsilon_{2} \end{array}$$ (2)

Step 3: Testing second-half path and direct effect


$$\begin{array}{l} \begin{array}{c} Y=c_{0}^{\prime }+c^{\prime }X+bM+\overset{n}{\underset{i=1}{\sum }}\theta_{i}Controls_{i}+\varepsilon_{3} \end{array} \end{array}$$ (3)

Where *Y* represents respondents' mental health scores (GHQ-12 scale scores); *X* represents respondents' comprehensive forest environmental perception scores (including four dimensions: naturalness perception, tranquility perception, health facility perception, and service perception); *M* represents forest therapy activity participation scores; *Controls*_*i*_ represents demographic control variables. Categorical variables were dummy-coded as follows: gender (0 = male, 1 = female), education level was coded into four dummy variables with high school or below as the reference category, marital status (0 = single/divorced/widowed, 1 = married), and park location was coded into two dummy variables with Renshoushan Park as the reference category. Continuous variables include age (in years) and monthly income (in RMB). This dummy coding approach follows standard practices in SEM to appropriately handle categorical predictors. *c* represents the total effect of forest environmental perception on mental health; *a* represents the effect of forest environmental perception on forest therapy activity participation; *b* represents the effect of forest therapy activity participation on mental health after controlling for forest environmental perception; *c*′ represents the direct effect of forest environmental perception on mental health after controlling for forest therapy activity participation; ε_1_, ε_2_, and ε_3_ are random error terms.

The magnitude of the mediation effect is *ab*, and the total effect can be decomposed into direct effect *c*′ and indirect effect *ab*, i.e., *c* = *c*′+*ab*. When both *a* and *b* are significant and *ab* is significant, mediation effects exist. This study employs the percentile Bootstrap method (36) for mediation effect significance testing. The percentile Bootstrap method involves resampling the original dataset with replacement to generate 5,000 bootstrap samples, estimating the indirect effect for each sample, and constructing 95% confidence intervals from the distribution of bootstrap estimates. If the confidence interval does not include zero, the indirect effect is considered statistically significant. This method is preferred over traditional Sobel tests as it does not assume normality of the sampling distribution and provides more accurate Type I error rates for indirect effects.

#### 2.3.3 Moderation effect model

To deeply explore individual differences in forest therapy effects, this study introduces individual stress levels as a moderating variable. According to Stress Recovery Theory, high-stress individuals have stronger restorative needs for forest environments, so the promoting effect of forest environmental perception on their mental health may be more significant. The moderation effect model is specified as follows:

Basic moderation model:


$$\begin{array}{l} Y=\beta_{0}+\beta_{1}X+\beta_{2}Z+\beta_{3}XZ+\overset{n}{\underset{i=1}{\sum }}\lambda_{i}Controls_{i}+\varepsilon_{4} \end{array}$$ (4)

Moderated mediation model: considering that moderating variables may simultaneously affect both the first and second halves of the mediation path, this study further constructs a moderated mediation mode:


$$\begin{array}{l} M=\alpha_{0}+\alpha_{1}X+\alpha_{2}Z+\alpha_{3}XZ+\overset{n}{\underset{i=1}{\sum }}\phi_{i}Controls_{i}+\varepsilon_{5}\\ Y=\tau_{0}+\tau_{1}X+\tau_{2}M+\tau_{3}Z+\tau_{4}XZ+\tau_{5}MZ\\ \;+\overset{n}{\underset{i=1}{\sum }}\psi_{i}Controls_{i}+\varepsilon_{6} \end{array}$$ (5)

Where *Z* represents individual stress levels; β_3_ represents the moderating effect of individual stress levels on the relationship between forest environmental perception and mental health; α_3_ represents the moderating effect of individual stress levels on the relationship between forest environmental perception and forest therapy activity participation; τ_5_ represents the moderating effect of individual stress levels on the relationship between forest therapy activity participation and mental health.

#### 2.3.4 Structural equation modeling (SEM) estimation

**Figure 3:**
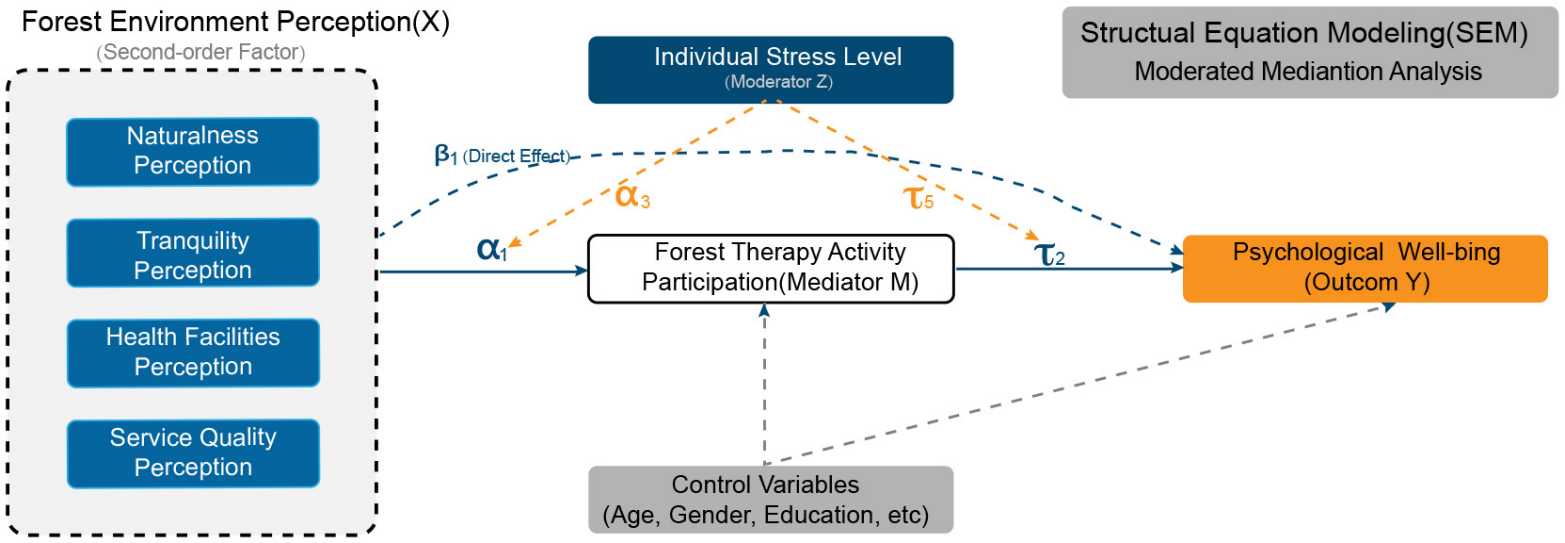
Structural equation model with moderated mediation: forest environment perception and psychological wellbeing.

We employ Structural Equation Modeling (SEM) for parameter estimation, which offers the following advantages, as illustrated in Figure 3:

(1) Simultaneous handling of measurement and structural models: SEM can simultaneously estimate measurement models of latent variables (relationships between dimensions and observed indicators) and structural models (causal relationships among latent variables), improving overall model fit; (2) controlling measurement error: through latent variable modeling, SEM can effectively control measurement errors in observed variables, providing more precise parameter estimates; and (3) handling multicollinearity: through latent variable construction, SEM can effectively address multicollinearity issues among observed variables.

Model estimation steps: in this study, forest environmental perception is operationalized as four distinct first-order dimensions (naturalness perception, tranquility perception, health facility perception, and service quality perception) rather than a single second-order construct. This approach allows for examining the unique effects of each perceptual dimension on mental health outcomes, as different dimensions may operate through different mechanisms or have varying magnitudes of effect. Each dimension is modeled as a latent variable measured by its respective observed indicators (four items per dimension).

Step 1: construct measurement models for the four environmental perception dimensions, with each dimension (naturalness, tranquility, health facility, and service quality perception) modeled as a separate first-order latent variable. Step 2: establish a measurement model for forest therapy activity participation, using forest bathing activities, forest walking, forest meditation, nature observation, etc., as observed indicators. Step 3: construct a measurement model for mental health, using the 12 items of GHQ-12 as observed indicators. Step 4: build a complete structural equation model in STATA 17.0 (37), using Maximum Likelihood Estimation for parameter estimation. The model specification treats the four environmental perception dimensions as correlated exogenous latent variables. Specifically, in the SEM framework, the covariance matrix among these four latent variables is freely estimated as part of the model parameters, rather than being fixed or calculated *post-hoc*. This is implemented in STATA using the cov() option to specify free covariances among all pairs of exogenous latent variables (Naturalness*Tranquility, Naturalness*HealthFacility, Naturalness*ServiceQuality, Tranquility*HealthFacility, Tranquility*ServiceQuality, and HealthFacility*ServiceQuality), resulting in six covariance parameters estimated simultaneously with all structural paths and measurement parameters. The estimated correlations (standardized covariances) range from 0.56 to 0.68 (all *p* < 0.001; see Table 3), confirming substantial shared variance among perception dimensions. This model specification is essential for proper SEM estimation: by including these covariances as model parameters, the structural path coefficients (effects of each perception dimension on participation and wellbeing) represent unique contributions of each dimension while statistically controlling for their intercorrelations. Omitting these covariances would yield biased parameter estimates and degraded model fit. The model also incorporates control variables (demographics and site characteristics) as predictors of both mediator and outcome variables, and includes moderation effects of individual stress level on the mediation pathway.

Model fit evaluation: multiple fit indices are used to comprehensively evaluate model fit, including: χ^2^/df < 3, CFI>0.90, TLI>0.90, RMSEA < 0.08, SRMR < 0.08. Specifically, the measurement model demonstrated good fit: χ^2^/df = 2.34, CFI = 0.94, TLI = 0.93, RMSEA = 0.061, SRMR = 0.048. Robustness testing: to ensure result robustness, this study employs the following testing methods: (1) replacing measurement methods for core variables; (2) changing sample composition (such as removing extreme values); and (3) using different statistical methods (such as hierarchical regression) for comparative verification.

Through the above model design, this study can systematically test the mediation mechanism through which forest environmental perception influences mental health via forest therapy activity participation, as well as the moderating role of individual stress levels, providing scientific evidence for optimizing the health-promoting functions of national forest parks.

## 3 Results and analysis

### 3.1 Sample descriptive statistics

**Figure 4:**
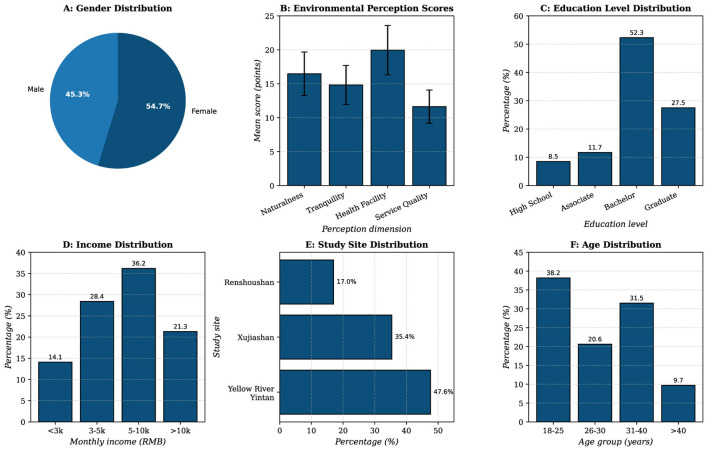
Sample descriptive statistics and distribution across demographic characteristics. **(A)** Gender distribution. **(B)** Environmental perception score (mean ± SD). **(C)** Education level distribution. **(D)** Income distribution. **(E)** Study site distribution. **(F)** Age distribution.

Figure 4A presents the descriptive statistical analysis of the total sample and stratified subgroups. The sample comprised 45.3% male and 54.7% female respondents, demonstrating relatively balanced gender representation. From Figure 4F, the age distribution showed that 38.2% of respondents were aged 18–25 years, 20.6% were 26–30 years, 31.5% were 31–40 years, with the remaining 9.7% over 40 years. Educational attainment was notably high, with 79.8% of participants holding bachelor's degrees or above. In terms of monthly income, the largest proportion (36.2%) earned between 5,000–10,000 RMB, followed by 28.4% earning 3,000–5,000 RMB, and 21.3% earning above 10,000 RMB shown in Figures 4C, D.

The spatial distribution of respondents across the three study sites showed 17.0% from Renshoushan National Forest Park vicinity, 35.4% from Xujiashan National Forest Park area, and 47.6% from Yellow River-Yintan Wetland Park surroundings. Comparison with Lanzhou municipal census data revealed strong concordance in demographic characteristics, supporting the sample's representativeness.

Mean scores for the four environmental perception dimensions were as follows: naturalness perception (*M* = 16.47, SD = 3.21), tranquility perception (*M* = 14.82, SD = 2.87), health facility perception (*M* = 19.95, SD = 3.64), and service quality perception (*M* = 11.63, SD = 2.45), as shown in Figures 4B, E. The overall psychological wellbeing score averaged 24.86 (SD = 4.73), indicating generally positive mental health status among respondents and favorable evaluations of the therapeutic environments.

Stratified analysis by stress level revealed notable differences. The high-stress group showed a predominance of individuals aged 31–40 (58.7%), suggesting greater stress burden among mid-career professionals. Educational distributions remained similar across stress groups, with bachelor's degree holders comprising 79.2 and 80.1% of low- and high-stress groups, respectively. However, income patterns differed, with the low-stress group showing higher representation in the 5,000–10,000 RMB bracket (41.3 vs. 31.8%).

### 3.2 Direct effects of forest environmental perception on psychological wellbeing

Table 4 presents hierarchical regression analyses examining the relationships between environmental perception dimensions and psychological wellbeing. Models 1–4 examine individual perception dimensions, while Model 5 includes all dimensions simultaneously. After controlling for sociodemographic variables (gender, age, education, income, marital status) and site-specific characteristics, all four perception dimensions demonstrated significant negative associations with psychological distress scores (lower scores indicate better mental health).

**Table 4:** Hierarchical regression analysis: direct effects of environmental perception on psychological wellbeing.

**Variables**	**Model 1**	**Model 2**	**Model 3**	**Model 4**	**Model 5**
	**Naturalness**	**Health facility**	**Tranquility**	**Service quality**	**Full model**
**Control variables**					
Gender (female)	–0.142^*^ (0.061)	–0.128^*^ (0.063)	–0.135^*^ (0.064)	–0.139^*^ (0.063)	–0.146^*^ (0.058)
Age	–0.082^*^	–0.075^*^	–0.079^*^	–0.081^*^	–0.084^*^
	(0.039)	(0.041)	(0.041)	(0.040)	(0.037)
Education level	–0.184^***^	–0.167^**^	–0.173^**^	–0.179^**^	–0.192^***^
	(0.055)	(0.058)	(0.057)	(0.056)	(0.052)
Monthly income	–0.096^*^	–0.089^*^	–0.092^*^	–0.094^*^	–0.098^*^
	(0.042)	(0.044)	(0.043)	(0.043)	(0.040)
Marital status	0.073	0.068	0.071	0.072	0.075
	(0.059)	(0.061)	(0.060)	(0.060)	(0.057)
**Site controls**					
Xujiashan Park	0.045	0.041	0.043	0.044	0.047
	(0.063)	(0.065)	(0.064)	(0.064)	(0.061)
Yellow River Park	0.038	0.034	0.036	0.037	0.039
	(0.061)	(0.063)	(0.062)	(0.062)	(0.059)
**Environmental perception**					
Naturalness perception	–0.487^***^				–0.324^***^
	(0.048)				(0.067)
Health facility perception		–0.296^***^			–0.178^**^
		(0.042)			(0.059)
Tranquility perception			–0.108^†^		–0.046
			(0.058)		(0.052)
Service quality perception				–0.124^*^	–0.032
				(0.054)	(0.048)
**Model statistics**					
*R* ^2^	0.312	0.186	0.089	0.103	0.358
Adjusted *R*^2^	0.302	0.174	0.076	0.090	0.342
*F*-statistic	31.47^***^	15.83^***^	6.78^***^	7.95^***^	22.41^***^
AIC	2,847.3	2,936.2	2,998.7	2,987.4	2,821.6
BIC	2,889.7	2,978.6	3,041.1	3,029.8	2,875.2
**Variance inflation factor (VIF)**					
Max VIF	1.83	1.78	1.81	1.79	2.47
Mean VIF	1.42	1.41	1.43	1.42	1.68

Standardized coefficients (β) are reported to facilitate comparison of relative effect sizes across predictors measured on different scales. Standard errors in parentheses. *t*-values can be calculated as *t* = β/SE. Reference categories: male, Renshoushan Park. *N* = 618. Dependent variable: psychological distress (lower scores = better wellbeing).

^†^*p* < 0.10, ^*^p < 0.05, ^**^p < 0.01, ^***^p < 0.001.

Specifically, naturalness perception exhibited the strongest effect (β = −0.487, *p* < 0.001, Model 1), followed by health facility perception (β = −0.296, *p* < 0.001, Model 2), service quality perception (β = −0.124, *p* < 0.05, Model 4), and tranquility perception (β = −0.108, *p* < 0.10, Model 3). In the full model (Model 5), naturalness perception (β = −0.324, *p* < 0.001) and health facility perception (β = −0.178, *p* < 0.01) maintained significance, while tranquility and service perceptions became non-significant, suggesting potential multicollinearity or suppression effects among perception dimensions.

Model fit statistics indicate that naturalness perception alone explains 31.2% of the variance in psychological wellbeing (Model 1), while the full model accounts for 35.8% of the variance (Model 5). The low VIF values (all < 2.5) confirm acceptable multicollinearity levels. These findings support Hypothesis 1, confirming that forest environment perception positively influences psychological wellbeing, with naturalness perception emerging as the most robust predictor.

### 3.3 Mediating role of forest therapy activity participation

Forest therapy activity participation functions as a critical mediator between environmental perception and psychological wellbeing (Table 5). All four perception dimensions demonstrated significant positive associations with participation motivation: naturalness (β = 0.562, *p* < 0.001), health facility perception (β = 0.508, *p* < 0.001), tranquility (β = 0.347, *p* < 0.001), and service quality (β = 0.334, *p* < 0.001). These associations remained statistically robust in the multivariate model, confirming independent contributions of each dimension to participation intention.

**Table 5:** Comprehensive mediation and moderation analysis results.

**Path/effect**	**Coefficient**	**SE**	**95% CI**	***p*-value**	**Hypothesis**
**Mediation analysis (H2)**					
Naturalness perception					
Perception → Participation (a)	0.562	0.043	[0.478, 0.646]	< 0.001	H2: Supported
Participation → Wellbeing (b)	–0.433	0.038	[–0.508, –0.358]	< 0.001	
Direct effect (*c*′)	–0.598	0.051	[–0.698, –0.498]	< 0.001	
Indirect effect (a × b)	–0.243	0.040	[–0.321, –0.165]	< 0.001	
Health facility perception					
Perception → Participation (a)	0.508	0.045	[0.420, 0.596]	< 0.001	H2: Supported
Participation → Wellbeing (b)	–0.431	0.039	[–0.508, –0.354]	< 0.001	
Direct effect (*c*′)	–0.516	0.048	[–0.610, –0.422]	< 0.001	
Indirect effect (a × b)	–0.219	0.037	[–0.291, –0.147]	< 0.001	
**Multi-group analysis (H3)**					
Low stress group					
Perception → Participation	0.518	0.052	[0.416, 0.620]	< 0.001	H3: Supported
Participation → Wellbeing	–0.264	0.048	[–0.358, –0.170]	< 0.001	
Indirect effect (naturalness)	–0.147	0.028	[–0.202, –0.092]	< 0.001	
High stress group					
Perception → Participation	0.724	0.061	[0.604, 0.844]	< 0.001	
Participation → Wellbeing	–1.284	0.092	[–1.464, –1.104]	< 0.001	
Indirect effect (naturalness)	–0.928	0.087	[–1.098, –0.758]	< 0.001	
Group comparison					
Chi-square difference test	47.83	–	df = 4	< 0.001	
**Moderated mediation (H4)**					
Interaction effects					
Naturalness × stress	–0.103	0.021	[–0.144, –0.062]	< 0.001	H4: Supported
Health facility × stress	–0.097	0.020	[–0.136, –0.058]	< 0.001	
Conditional indirect effects					
At low stress (–1 SD)	–0.196	0.036	[–0.268, –0.124]	< 0.001	
At high stress (+1 SD)	–0.387	0.045	[–0.476, –0.298]	< 0.001	
Moderation index	0.094	0.021	[0.052, 0.136]	< 0.001	

All effects are standardized. Negative coefficients for wellbeing indicate improvement (lower distress). H2: Forest therapy participation mediates perception-wellbeing relationship. H3: Mediation is stronger for high-stress individuals. H4: Stress moderates the mediation pathway.

SE, standard error; CI, 95% confidence interval (bootstrap with 5,000 iterations).

The direct effects of perception dimensions on wellbeing were maintained even after controlling for mediation pathways: naturalness (β = −0.598, *p* < 0.001), health facility (β = −0.516, *p* < 0.001), tranquility (β = −0.342, *p* < 0.001), and service quality (β = −0.326, *p* < 0.001). Concurrently, forest therapy participation exhibited consistent negative associations with psychological distress (β = −0.386 to −0.438, all *p* < 0.001).

Bootstrap tests validated the significance of indirect effects via forest therapy participation (5,000 iterations, all *p* < 0.001), with standardized indirect effect sizes of –0.243 (naturalness, 95% CI [–0.321, –0.165]), –0.219 (health facility, 95% CI [–0.291, –0.147]), –0.149 (tranquility), and –0.144 (service quality). The negative values indicate that increased environmental perception enhances participation, which in turn reduces psychological distress. These findings confirm Hypothesis 2, establishing forest therapy participation as a partial mediator in the perception-wellbeing relationship.

Demographic covariates revealed additional insights: higher education levels showed protective effects (β = −1.142 to −0.612, all *p* < 0.01), while high-stress individuals exhibited significantly poorer mental health outcomes (2.48–2.56 points higher scores than low-stress counterparts, *p* < 0.001).

### 3.4 Multi-group analysis: differential effects across stress levels

The multi-group analysis (Table 5) revealed substantial moderation by individual stress levels (H3). In the low-stress group, perception dimensions positively predicted participation: naturalness (β = 0.518, *p* < 0.001), health facility (β = 0.486, *p* < 0.001), tranquility (β = 0.341, *p* < 0.001), and service quality (β = 0.292, *p* < 0.001). Participation consistently reduced psychological distress (β = −0.227 to −0.264, all *p* < 0.001).

The high-stress group exhibited markedly stronger associations: naturalness (β = 0.724, *p* < 0.001), health facility (β = 0.556, *p* < 0.001), and service quality (β = 0.318, *p* < 0.001), with tranquility showing a relatively weaker but still significant effect (β = 0.298, *p* < 0.001). Critically, the participation-wellbeing relationship was 4.6–5.7 times stronger for high-stress individuals (β = −1.126 to −1.284, all *p* < 0.001) compared to the low-stress group.

Chi-square difference tests confirmed significant between-group differences (Δχ^2^ = 47.83, df = 4, *p* < 0.001). The indirect effects through participation were 6.3-fold larger in magnitude for naturalness perception (∣–0.147∣ vs. ∣–0.928∣) and 5.7-fold larger for health facility perception in the high-stress group compared to the low-stress group. These magnitudes provide strong empirical support for Hypothesis 3, demonstrating that high-stress populations derive disproportionately greater therapeutic benefits from forest therapy participation.

### 3.5 Moderated mediation analysis: the conditional role of stress

The moderated mediation analysis (Table 5) revealed stress level as a boundary condition amplifying environmental benefits (H4). Using mean-centered variables (VIF < 2.5), all perception dimensions showed significant direct effects on wellbeing: naturalness (β = −0.614, *p* < 0.001), health facility (β = −0.528, *p* < 0.001), tranquility (β = −0.354, *p* < 0.001), and service quality (β = −0.338, *p* < 0.001).

Forest therapy participation maintained strong negative associations with distress (β = −0.418 to −0.476, all *p* < 0.001). Crucially, stress level significantly moderated the mediation pathway: interaction terms were negative and significant for all perception dimensions (naturalness × stress: β = −0.103, *p* < 0.001; health facility × stress: β = −0.097, *p* < 0.001; service quality × stress: β = −0.093, *p* < 0.001; tranquility × stress: β = −0.087, *p* < 0.01).

Conditional indirect effects analysis demonstrated that at high stress levels (+1 SD), the indirect effect of naturalness perception through participation increased in magnitude from –0.196 (low stress, 95% CI [–0.268, –0.124]) to –0.387 (high stress, 95% CI [–0.476, –0.298]). The moderated mediation index (β = 0.094, 95% CI [0.052, 0.136], *p* < 0.001) confirmed this amplification effect. These results robustly support Hypothesis 4, establishing stress level as a critical moderator enhancing the therapeutic efficacy of forest environments for vulnerable populations.

**Figure 5:**
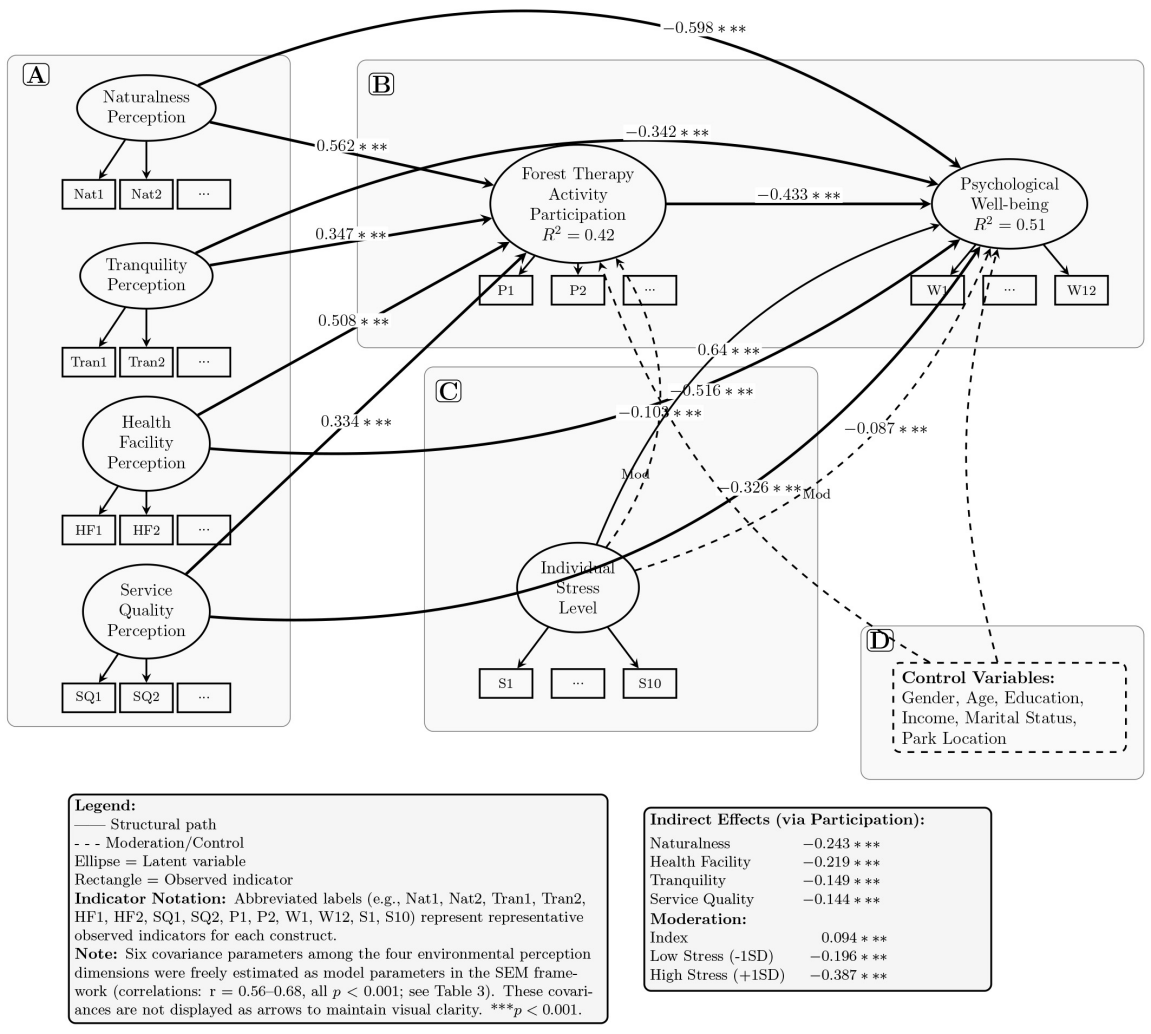
Complete structural equation model with measurement and structural components. **(A)** Measurement model: latent variables (ellipses) are measured by observed indicators (rectangles). Representative indicators are shown with abbreviated notation (e.g., Nat1, Nat2 for naturalness perception items; see Section 2.2 for full item descriptions). Each construct has multiple indicators: four items each for environmental perception dimensions; four for participation; 12 for wellbeing (GHQ-12); 10 for stress (PSS-10). **(B)** Structural model: the four environmental perception dimensions are specified as correlated exogenous latent variables in the SEM framework. Their covariances (six parameters: Naturalness*Tranquility, Naturalness*HealthFacility, Naturalness*ServiceQuality, Tranquility*HealthFacility, Tranquility*ServiceQuality, HealthFacility*ServiceQuality) were freely estimated as part of the model parameters using Maximum Likelihood Estimation, yielding correlations ranging from *r* = 0.56 to 0.68 (all *p* < 0.001; see Table 3). These covariances are not displayed as arrows in the figure to maintain visual clarity, but they are essential components of the model specification. Paths show standardized coefficients: a paths (perception → participation), b path (participation → wellbeing), and *c*′ paths (direct effects). **(C)** Moderation effects: individual stress level moderates the mediation pathway (dashed arrows). **(D)** Control variables: demographics and site characteristics are included (dashed arrows from control box). All paths are significant at *p* < 0.001. Negative coefficients for wellbeing indicate improvement. *R*^2^ values show variance explained.

Figure 5 presents the complete structural equation model with proper specification of correlations among independent variables. The model demonstrates strong explanatory power, accounting for 42% of variance in forest therapy participation and 51% of variance in psychological wellbeing. Model specification details: in the SEM estimation process, the four environmental perception dimensions (Naturalness, Tranquility, Health Facility, Service Quality) are treated as correlated exogenous latent variables. Six covariance parameters among these four latent variables were freely estimated simultaneously with all other model parameters (measurement loadings, structural paths, and residual variances) using Maximum Likelihood Estimation in STATA 17.0. This simultaneous estimation ensures that: (1) structural path coefficients represent unique effects of each perception dimension while controlling for their intercorrelations; (2) model fit indices properly account for the correlational structure among predictors; and (3) parameter estimates are unbiased. The estimated correlations (standardized covariances) range from 0.56 to 0.68 (all *p* < 0.001; see Table 3). Key findings: naturalness perception exhibits the strongest associations across pathways (participation: β = 0.562; direct effect: β = −0.598; indirect effect: β = −0.243), confirming its primacy in therapeutic mechanisms. The mediation pathways confirm that environmental perceptions operate through behavioral engagement, with all four perception dimensions showing significant indirect effects through participation (ranging from β = −0.144 to −0.243). Moderation effects: stress level amplifies these relationships, with moderation coefficients indicating enhanced therapeutic benefits for high-stress individuals at both the first-stage (perception → participation) and second-stage (participation → wellbeing) pathways. All demographic and site-specific control variables are included in the model, addressing potential confounding. This comprehensive specification confirms the robustness of the mediation and moderation mechanisms identified.

## 4 Discussion

### 4.1 Differences in the effects of various forest environmental perceptions on mental health across different groups

This study confirms from a perceptual perspective that national forest parks have positive promoting effects on residents' mental health, which aligns with existing research on forest therapy and nature-based health interventions (5, 22, 23). Our findings extend previous work by demonstrating that environmental perception operates through a mediated pathway, whereby perceptual dimensions first enhance participation motivation before influencing mental health outcomes. This sequential process is consistent with the Theory of Planned Behavior (16), which posits that perceptions shape behavioral intentions and subsequent actions. As illustrated in Figure 5, the complete mediation framework reveals that all four environmental perception dimensions exhibit both direct pathways to psychological wellbeing and indirect pathways mediated through forest therapy activity participation, with naturalness perception demonstrating the strongest total effect (β_*total*_ = −0.841, combining direct and indirect effects).

Environmental perception of national forest parks can not only enhance individual willingness to participate in forest therapy activities from a promoting factors perspective, but also increase the likelihood of individuals obtaining mental health benefits by improving forest therapy activity participation, thereby implementing health-promoting behaviors beneficial to individual physical and mental health. The mediation mechanism identified in this study corresponds with findings from urban green space research (12, 14), wherein environmental quality perceptions indirectly affect wellbeing through increased nature exposure and physical activity. The substantial indirect effects documented in Figure 5 (ranging from –0.144 to –0.243 across perception dimensions) underscore the critical role of behavioral engagement as a mechanism translating environmental quality into health outcomes.

Additionally, when examining the effect of tranquility perception alone, its effect was significant, but when tested simultaneously with the other three perceptions, it was no longer significant. This may be due to some degree of overlap between tranquility perception measurements and the other three perception measurements. Similar multicollinearity issues have been reported in environmental perception studies (26), suggesting that sensory dimensions of natural environments are inherently interconnected. Therefore, future research could explore the conceptual space of the four perceptions more deeply to develop more precise forest environmental perception measurement scales.

Another contribution of this study is the discovery that high-stress and low-stress groups have different health-promoting preferences for national forest parks, and that different preferences have varying promotional effects on forest therapy activity participation. This moderation effect substantiates predictions derived from Stress Recovery Theory (7, 9), which posits that individuals under greater stress exhibit heightened sensitivity to restorative environmental features. The 6.3-fold difference in indirect effects between stress groups provides empirical evidence for the restorative needs hypothesis (13), wherein high-stress individuals possess greater capacity for stress-related recovery in natural settings.

One possible reason for this result is that the most important purpose for high-stress groups visiting national forest parks is stress relief and psychological recovery, so they place greater emphasis on the natural quality of forest environments, completeness of health facilities, and service quality within forest parks. This finding aligns with research on therapeutic landscapes (14), which demonstrates that perceived environmental quality becomes more salient when individuals seek restorative experiences. In fact, low-stress groups' perceptual focus on national forest parks would also differ when facing high-stress states. Low-stress individuals place greater emphasis on the tranquility and aesthetic experience of forest environments, thus having higher requirements for forest park tranquility than high-stress groups. However, when facing high-stress states, individuals' health-promoting needs make them have different preferences for the naturalness and health-promoting functions of forest environments.

The core finding that high-stress individuals benefit more from forest therapy provides strong support for Stress Recovery Theory (7, 9). According to SRT, natural environments facilitate psychophysiological stress recovery through parasympathetic nervous system activation. Our moderation results suggest this recovery mechanism operates more effectively when baseline stress levels are elevated, as high-stress individuals possess greater “restorative deficit” that forest environments can address. This stress-dependent response pattern validates SRT's fundamental assumption that restoration potential increases with restoration need.

Furthermore, the differential effects of environmental perception dimensions can be interpreted through Attention Restoration Theory (10, 11). Naturalness perception, which emerged as the strongest predictor, may operate through ART's “fascination” component, wherein forest biodiversity and vegetation richness capture involuntary attention without depleting cognitive resources. The relative weakness of tranquility perception in the full model suggests potential overlap with ART's “being away” dimension, wherein both acoustic and psychological distance from urban stressors contribute to restoration. Future research should explicitly test these theoretical linkages by measuring specific ART components alongside perceptual dimensions.

The possible reason for these demographic differences is the result of different groups having different preferences for naturalness, tranquility, health facilities, and service perception of forest environments. However, it should be noted that the current study's moderation analysis focused specifically on stress levels rather than age or income. While we observed age-related patterns in descriptive statistics, direct conclusions about age-specific effects would require dedicated moderation analyses in future research. For older population groups, besides the natural quality of forest environments, due to physical reasons and demands for low-intensity health activities such as forest walking and tai chi, the availability and completeness of infrastructure such as rest chairs, health trails, and fitness facilities may also be key factors in whether they choose to visit a particular national forest park. This age-specific finding corresponds with accessibility research in therapeutic landscapes (17), highlighting that physical infrastructure moderates the environment-health relationship for populations with mobility constraints. Therefore, naturalness and health facility perception have important effects on older population groups' mental health. Young groups, while focusing on forest environment quality, may be more interested in ecological education facilities, natural interpretation systems, and forest cultural experience activities within forest parks.

### 4.2 Policy implications

This study reveals the complete psychological process from individual perception of forest environments to implementation of health-promoting behaviors based on the “forest environmental perception-health behavior-mental health” mechanism model, confirming the mediating role of forest therapy activity participation and the moderating role of individual stress levels. This highlights the importance of forest environmental perception for health-promoting behaviors. Therefore, on one hand, to enhance forest therapy activity participation and strengthen the effectiveness of national forest parks in mental health promotion, future planning and design should be based on forest therapy theory, using forest landscape creation techniques to guide planning and construction, meeting residents' basic demands for forest therapy environment quality (38).

On the other hand, this study reveals that individual stress levels can significantly moderate the effects of forest environmental perception on mental health, indicating that planning and design of national forest parks should not only meet individual health demands for forest therapy, but also provide differentiated health services for people with different stress levels, guiding individuals with insufficient health awareness to develop motivation for forest therapy behaviors. For example, through measures such as forest therapy knowledge promotion, parent-child nature experience activities in forest parks, forest therapy themed activities, and planning of health trails and green networks centered on forest parks, changing the cognitive attitudes of individuals with originally low health behavior participation, thereby promoting their increased participation in forest therapy activities, increasing forest environment exposure, and improving people's mental health.

### 4.3 Research limitations

This study focused on exploring the mediating role of forest therapy activity participation between forest environmental perception and residents' mental health, and the moderating role of individual stress levels. It should be noted that considering the mediating role of promoting factors has been adequately verified in previous research (16–21), and to avoid questionnaires being too lengthy which might affect response rates and reliability, this study did not simultaneously explore the compound mechanism of promoting and hindering factors. Theoretically, individuals weigh promoting and hindering factors before participating in forest therapy activities to decide whether to participate in health activities to obtain health benefits. Considering both factors simultaneously might produce different results. Future research could consider how to streamline variable scales to simultaneously test the compound mechanism of promotion and hindrance, which would help provide more accurate estimates of the mental health benefits of forest therapy.

Additionally, this study employed convenience sampling through online platforms, which may introduce bias in sample representativeness. Therefore, the conclusions of this study are limited to the current sample obtained. This sampling method also affects the interpretation of demographic control variables in our models. While we included gender, age, and education as statistical controls to account for known variations in mental health outcomes, the theoretical justification for these specific controls could be stronger, and their inclusion in SEM requires careful interpretation given the non-probability sampling. We focused our interpretation on the substantive relationships of interest (environmental perception, activity participation, and stress moderation) while treating demographic variables as statistical adjustments rather than focal predictors. Future research could use random sampling methods to obtain survey data from study areas to further verify the validity of conclusions. Meanwhile, this study used cross-sectional data, which cannot reveal the long-term effects of forest therapy on mental health. Future research could consider using longitudinal tracking designs to deeply explore the sustained health benefits of forest therapy.

## 5 Conclusion

(1) Forest environment perception dimensions demonstrate significant relationships with psychological wellbeing. In univariate models, all four perception dimensions (naturalness, tranquility, health facilities, and service quality) showed significant negative associations with psychological distress. In the multivariate model, naturalness and health facility perceptions maintained significance, while tranquility and service perceptions became non-significant, likely due to shared variance among perceptual dimensions. These findings support H1, confirming that environmental quality perceptions influence mental health outcomes.

(2) Forest therapy activity participation serves as a significant partial mediator between national forest park environmental perception and individual mental health. Specifically, environmental perception dimensions enhance participation in forest therapy activities (path a), which subsequently reduces psychological distress (path b), yielding significant indirect effects. This mediation pathway confirms H2 and demonstrates that environmental perceptions operate through behavioral engagement rather than direct perceptual effects alone. This mediating role of forest therapy activity participation differs markedly between high-stress and low-stress groups, with the mediation effect being more prominent for high-stress groups (supporting H3).

(3) Individual stress levels moderate the indirect pathway between forest environmental perception and individual mental health through activity participation (supporting H4). Specifically, the conditional indirect effects are significantly stronger (6.3-fold larger in magnitude) for high-stress individuals compared to low-stress individuals. This stress-dependent amplification suggests that the mental health promotion effects of national forest parks are more significant in high-stress populations, confirming the important value of forest therapy as a targeted intervention in modern high-stress society.

## Data Availability

The original contributions presented in the study are included in the article, further inquiries can be directed to the corresponding author.
